# The Toxicity of Heavy Metals and Chemical Pollutants in Agricultural Soil and Plants: Ecological Risks and Remediation

**DOI:** 10.3390/toxics14090805

**Published:** 2026-09-10

**Authors:** Peipei Song

**Affiliations:** College of Resources and Environment, Shandong Agricultural University, Tai’an 271018, China; songpeipei@sdau.edu.cn

## 1. Introduction

Accelerated industrialization and intensive agricultural practices have driven widespread accumulation of heavy metals and chemical pollutants in global agricultural soils [1,2]. These contaminants not only degrade soil ecological function and reduce crop productivity, but also enter the food chain via plant uptake, posing cascading threats to ecosystem health and human well-being [3,4]. Addressing these intertwined challenges demands integrated research on contaminant behavior, ecological risk quantification, and green remediation technologies.

This Special Issue (SI), entitled “The Toxicity of Heavy Metals and Chemical Pollutants in Agricultural Soil and Plants: Ecological Risks and Remediation”, was launched in *Toxics* to assemble state-of-the-art investigations into the occurrence, migration, ecotoxicology, detection, and remediation of contaminants in soil–plant systems. The SI received broad attention from the global research community, with 13 high-quality papers finally published, including 10 original research articles and three comprehensive reviews. Collectively, the contributions span regional contamination assessment, remediation technology development, phytotoxicity mechanisms, and microbial degradation, offering valuable insights for agricultural soil pollution control and sustainable land management.

## 2. Overview of Published Articles

The published papers are organized into three thematic clusters aligned with the core logic of environmental pollution research: contamination status investigation, remediation technology development, and toxic effects and risk evaluation.

### 2.1. Heavy Metal Contamination Assessment, Spatial Distribution and Source Identification

Reliable contamination evaluation and source apportionment are the foundations of targeted soil pollution management. Four studies employed multi-method approaches to assess heavy metal pollution across diverse land use types and regions.

Pan et al. (contribution 1) investigated soil heavy metals in sorghum cultivation bases of the Chishui River Basin, the core production region of Chinese sauce-aroma Baijiu. Based on 172 surface soil samples, they found notable enrichment of Cd and Hg, identified three major pollution sources via the positive matrix factorization (PMF) model, and confirmed Cd as the primary contributor to ecological risk. Ren et al. (contribution 6) focused on the Fengfeng Mining Area with a 150-year mining history, revealing that Cd, Cu, Pb and Zn exceeded local background values by 1.61-6.48 times, with Cd and Pb presenting high ecological risk based on chemical speciation analysis. Gruszka et al. (contribution 7) combined chemical analysis with the Ostracodtoxkit ecotoxicological test for urban garden soils in Poland, demonstrating that total metal concentrations overestimate actual environmental risk, and that Pb is the dominant driver of test organism growth inhibition. Alzahrani et al. (contribution 10) integrated GIS mapping and multivariate statistics to evaluate soil heavy metal hazards in southwestern Saudi Arabia, delineating spatial distribution patterns and classifying 36.58% of the study area as heavily contaminated.

### 2.2. Remediation Technologies and Materials for Contaminated Soil

Developing efficient, eco-friendly and cost-effective remediation technologies is a core priority for soil pollution control. The published studies cover two major technical pathways: physicochemical stabilization for heavy metals and microbial degradation for organic chemical pollutants.

#### 2.2.1. Physicochemical Stabilization of Heavy Metals

In situ immobilization with functional amendments is a widely applied strategy for heavy-metal-contaminated farmland. Qiu et al. (contribution 2) conducted a field-scale study on severely Cd-contaminated farmland, verifying that layered double hydroxides (LDHs) effectively reduce Cd bioavailability by elevating soil pH and transforming Cd from active to stable fractions. The amendment significantly decreased Cd accumulation in Artemisia argyi (a non-food crop) and reduced Cd release during moxa combustion, providing a practical strategy for safe utilization of heavily contaminated farmland. Han et al. (contribution 9) systematically compared iron- and manganese-modified activated carbon and biochar for As- and Sb-co-contaminated soil. The results showed that 3% FeMn-modified biochar achieved the optimal immobilization performance, and all modified carbon materials improved soil enzyme activities. In a comprehensive review, Xu et al. (contribution 13) summarized the preparation, modification and remediation mechanisms of straw biochar for heavy-metal-contaminated soil, and highlighted low-cost modified biochar and combined application with other amendments as key development directions.

#### 2.2.2. Microbial Degradation and Bioremediation of Chemical Pollutants

Microbial remediation represents a sustainable approach for eliminating organic chemical pollutants in soil. Morales-Olivares et al. (contribution 4) characterized the paraquat resistance and degradation capacity of Caballeronia zhejiangensis CEIB S4-3. The strain removed 40.8% of paraquat at an initial concentration of 12 mg/L within 48 h, and genomic analysis identified key genes involved in paraquat resistance and degradation, supporting its application in bioremediation. Chowdhury et al. (contribution 12) provided a comprehensive overview of microbial degradation of herbicide residues in Australian agricultural soils, summarizing dominant degrading strains, environmental influencing factors, and recent advances in omics-driven mechanism research.

### 2.3. Pollutant Uptake, Phytotoxicity and Food-Chain Health Risk

Elucidating pollutant transfer in soil–plant systems and associated health risks is critical for safeguarding food safety and ecological health.

Tang et al. (contribution 3) quantitatively assessed how interregional food trade modulates population exposure to soil-derived heavy metals via crop consumption in China. Their findings indicated that food trade markedly redistributes dietary risks: the central and northeast regions contribute up to 36.78% and 45.08% of dietary As and Cd intake, respectively, to other regions through rice trade. Rafikova et al. (contribution 5) explored the combined effects of salinization, oil contamination and heavy metals on soil biological activity and phytoremediation performance of oat and lupine. They identified catalase, urease and phosphatase as the most sensitive soil enzymes, and confirmed both plant species can degrade 33-46% of petroleum hydrocarbons under complex pollution conditions. Zhan et al. (contribution 8) investigated the roles of metal tolerance proteins (MTPs) in rice under gallium stress, an emerging high-tech pollutant, revealing the trade-off between mineral element homeostasis and heavy metal detoxification at the molecular level. Additionally, Singh et al. (contribution 11) presented a systematic review on heavy metal toxicity in cereals, elaborating uptake mechanisms, physiological impacts and mitigation strategies to support cereal safety production.

## 3. Future Perspectives and Concluding Remarks

The 13 contributions in this SI advance our understanding of heavy metal and chemical pollutant toxicity, ecological risk, and remediation in agricultural soil–plant systems. Nevertheless, several critical research gaps remain to be addressed.

First, research on emerging contaminants and combined pollution in agricultural soils needs to be advanced. Current studies predominantly target traditional heavy metals and conventional herbicides. Future work should pay greater attention to emerging pollutants such as microplastics, antibiotics, and PFAS in farmland soils. It is urgent to elucidate their co-occurrence, interactive toxicity, and co-transport mechanisms with heavy metals and to develop targeted remediation technologies for complex mixed contamination.

Second, mechanistic understanding and long-term field validation of remediation technologies should be enhanced. Future studies should harness multi-omics approaches to decipher plant–microbe–pollutant interactions and clarify the molecular regulatory networks governing pollutant detoxification and collaborative remediation. In parallel, long-term field monitoring is essential to systematically assess the stability, ecological safety, and economic viability of diverse remediation materials and technologies under realistic farming conditions, thereby paving the way for large-scale practical application.

Third, refined, systemic frameworks for ecological and human health risk assessment should be developed. Future risk evaluation should holistically integrate pollutant bioavailability, climatic variability, agricultural management practices, and interregional food trade networks. Establishing a full-chain risk prediction and early-warning system will enable precise soil pollution control and strengthen food safety governance from local to global scales.

Overall, this Special Issue compiles recent advances and new perspectives on agricultural soil pollution and remediation. We hope this SI will inspire further high-quality research and practical innovations in soil environmental protection and sustainable remediation.

## Data Availability

No new data were created or analyzed in this study. Data sharing is not applicable to this article.
